# Differential Modulation of Innate Immune Responses in Human Primary Cells by Influenza A Viruses Carrying Human or Avian Nonstructural Protein 1

**DOI:** 10.1128/JVI.00999-19

**Published:** 2019-12-12

**Authors:** Paula L. Monteagudo, Raquel Muñoz-Moreno, Miguel Fribourg, Uma Potla, Ignacio Mena, Nada Marjanovic, Boris M. Hartmann, Stuart C. Sealfon, Adolfo García-Sastre, Irene Ramos, Ana Fernández-Sesma

**Affiliations:** a Department of Microbiology, Icahn School of Medicine at Mount Sinai, New York, New York, USA; b Department of Medicine Division of Infectious Diseases, Icahn School of Medicine at Mount Sinai, New York, New York, New York, USA; c Department of Neurology, Icahn School of Medicine at Mount Sinai, New York, New York, USA; d Global Health and Emerging Pathogens Institute, Icahn School of Medicine at Mount Sinai, New York, New York, USA; e The Graduate School of Biomedical Sciences, Icahn School of Medicine at Mount Sinai, New York, New York, USA; Hudson Institute of Medical Research

**Keywords:** NS1 protein, dendritic cells, epithelial cells, influenza, innate immunity, interferons

## Abstract

The influenza A virus (IAV) nonstructural protein 1 (NS1) contributes to disease pathogenesis through the inhibition of host innate immune responses. Dendritic cells (DCs) release interferons (IFNs) and proinflammatory cytokines and promote adaptive immunity upon viral infection. In order to characterize the strain-specific effects of IAV NS1 on human DC activation, we infected human DCs with a panel of recombinant viruses with the same backbone (A/Puerto Rico/08/1934) expressing different NS1 proteins from human and avian origin. We found that these viruses induced a clearly distinct phenotype in DCs. Specifically, viruses expressing NS1 from human IAV (either H1N1 or H3N2) induced higher levels of expression of type I (IFN-α and IFN-β) and type III (IFN-λ1 to IFNλ3) IFNs than viruses expressing avian IAV NS1 proteins (H5N1, H7N9, and H7N2), but the differences observed in the expression levels of proinflammatory cytokines like tumor necrosis factor alpha (TNF-α) or interleukin-6 (IL-6) were not significant. In addition, using imaging flow cytometry, we found that human and avian NS1 proteins segregate based on their subcellular trafficking dynamics, which might be associated with the different innate immune profile induced in DCs by viruses expressing those NS1 proteins. Innate immune responses induced by our panel of IAV recombinant viruses were also characterized in normal human bronchial epithelial cells, and the results were consistent with those in DCs. Altogether, our results reveal an increased ability of NS1 from avian viruses to antagonize innate immune responses in human primary cells compared to the ability of NS1 from human viruses, which could contribute to the severe disease induced by avian IAV in humans.

**IMPORTANCE** Influenza A viruses (IAVs) cause seasonal epidemics which result in an important health and economic burden. Wild aquatic birds are the natural host of IAV. However, IAV can infect diverse hosts, including humans, domestic poultry, pigs, and others. IAVs circulating in animals occasionally cross the species barrier, infecting humans, which results in mild to very severe disease. In some cases, these viruses can acquire the ability to be transmitted among humans and initiate a pandemic. The nonstructural 1 (NS1) protein of IAV is an important antagonist of the innate immune response. In this study, using recombinant viruses and primary human cells, we show that NS1 proteins from human and avian hosts show intrinsic differences in the modulation of the innate immunity in human dendritic cells and epithelial cells, as well as different cellular localization dynamics in infected cells.

## Abstract (precis)

Influenza A viruses (IAVs) cause seasonal epidemics which result in an important health and economic burden. Wild aquatic birds are the natural host of IAV. However, IAV can infect diverse hosts, including humans, domestic poultry, pigs, and others. IAVs circulating in animals occasionally cross the species barrier, infecting humans, which results in mild to very severe disease. In some cases, these viruses can acquire the ability to be transmitted among humans and initiate a pandemic. The nonstructural 1 (NS1) protein of IAV is an important antagonist of the innate immune response. In this study, using recombinant viruses and primary human cells, we show that NS1 proteins from human and avian hosts show intrinsic differences in the modulation of the innate immunity in human dendritic cells and epithelial cells, as well as different cellular localization dynamics in infected cells.

## INTRODUCTION

The successful recovery from viral infection largely depends on efficient activation of innate and adaptive immune responses. The activation of the innate immune response, especially interferon (IFN) responses, is an important mechanism for controlling virus replication and represents an early barrier against viruses such as influenza A virus (IAV). Mammalian cells have developed sophisticated antiviral mechanisms based on sensing viral products and triggering of signaling cascades leading to secretion of type I IFN (IFN-α and IFN-β), type III IFN (IFN-λs), and proinflammatory cytokines that inhibit viral replication and contribute to the initiation of more specific adaptive immune responses (1). Specifically, IAV double-stranded RNA (dsRNA) is recognized by Toll-like receptor 3 (TLR3), and IAV single-stranded RNA (ssRNA) is recognized by TLR7, two endosome membrane-associated pattern recognition receptors (PRRs) (2–4). Viral RNA products are recognized by cytosolic receptors such as the retinoic acid-inducible gene I (RIG-I) and RIG-I-like receptors (RLRs) (5). Sensing results in the activation of transcription factors, such as IFN regulatory factor 3 (IRF3), IRF7, and nuclear factor kappa B (NF-κB), that are responsible for the initiation of the transcription of type I/III IFNs and proinflammatory cytokines (1, 6, 7). After secretion, IFNs act in a paracrine and/or autocrine way to induce the expression of IFN-stimulated genes (ISGs), many of which have antiviral activity.

Viruses need to evade innate immunity in order to successfully replicate, establish infection, and ultimately be transmitted to other hosts. The nonstructural protein 1 (NS1) of IAV is a multifunctional protein able to antagonize host immune responses. It is highly expressed in the cytoplasm and the nucleus of infected cells and interacts with many cellular and viral proteins counteracting cellular defenses, especially the IFN responses of the host (8–10). NS1 has two distinct functional domains: an N-terminal RNA binding domain (RBD) (residues 1 to 73) and a C-terminal effector domain (ED) (from residue 85 to the end), separated by a short linker region. The last amino acids (from residue 207 to the end) form a disordered and flexible tail, which is one of the most variable regions among different NS1 proteins (11–13).

Different mechanisms have been reported by which the influenza NS1 protein counteracts the innate immune responses (reviewed in references 10 and 14). Briefly, NS1 can interact with different components of the RIG-I/IFN signaling pathway to efficiently inhibit IFN expression (reviewed in reference 15) either by direct binding of NS1 to RIG-I (16, 17) or by interaction with the E3 ligase tripartite motif-containing protein 25 (TRIM25). In addition to that, NS1 can also interact with Riplet, preventing ubiquitination and activation of RIG-I (18, 19). NS1 was also shown to inhibit IRF3 and NF-κB activation, which is necessary for the production of IFNs in infected cells (20, 21). The antiviral properties of protein kinase R (PKR) and 2′,5′-oligo(A) synthetase (OAS)-RNase L are also inhibited by NS1 as a result of the direct interaction with dsRNAs (22–25) and, in the case of PKR, also in a dsRNA binding-independent manner (22, 26, 27). In addition, NS1 proteins from some IAV strains can suppress the expression of cellular genes by blocking the maturation of cellular pre-mRNA through the interaction with the 30-kDa subunit of the cleavage and polyadenylation specificity factor (CPSF30) (28–31) and with the poly(A)-binding protein II (PABPII) (32). Also, NS1 blocks the export of cellular mRNA from the nucleus to the cytoplasm (33, 34), leading to a general inhibition of expression of the host’s genes, including IFN genes, ISGs, and proinflammatory genes. In addition to counteracting the innate immune response, NS1 can also target some factors (eIF4GI, PABPI, and human Staufen [hStaufen]) to enhance viral mRNA translation in the cytoplasm (35–37), and NS1 is also believed to be involved in regulation of the activation of phosphatidylinositol 3-kinase (PI3K) by binding p85β subunit (38, 39) and limiting the apoptosis of the cell (40). Furthermore, since some functions are carried out in the nucleus and some in the cytoplasm, the localization of NS1 is highly regulated through two nuclear localization signals (NLS) (41) and one nuclear export signal (NES) (42). Importantly, most of these NS1 functions are not conserved among different strains (reviewed in reference 43).

Immune cells, such as macrophages and dendritic cells (DCs), are crucial for the innate immune response against viruses and other pathogens. DCs express high levels of PRRs, which allows them to efficiently sense pathogen-associated molecular patterns (PAMPs). Upon sensing, DCs get activated and efficiently produce proinflammatory cytokines, IFNs, and chemokines, inducing an antiviral response in neighboring cells. Also, since they are professional antigen-presenting cells (APCs), they can process the antigens and present them by the major histocompatibility complex (MHC) to the T cells, inducing the initiation of long-term adaptive immunity. We have previously shown that the NS1 protein from IAV counteracts the production of type I IFN and the activation of human DCs (44–47). Due to the intrinsic variability of the NS1 proteins among different strains, we hypothesized that the modulation of the innate immune responses in DCs by NS1 during IAV infection is strain specific. Using recombinant IAVs expressing NS1 proteins of different origins (human or avian IAV) in an isogenic backbone, we found that infection with viruses expressing NS1 from a human or avian IAV results in different patterns of innate immune responses and expression of viral mRNA in DCs and in human primary epithelial cells. Furthermore, imaging flow cytometry experiments suggest that these differences could be associated with differential cellular localization dynamics of the NS1 proteins from different hosts.

## RESULTS

### Characterization of recombinant A/Puerto Rico/08/34 influenza viruses expressing different NS1 proteins.

**FIG 1:**
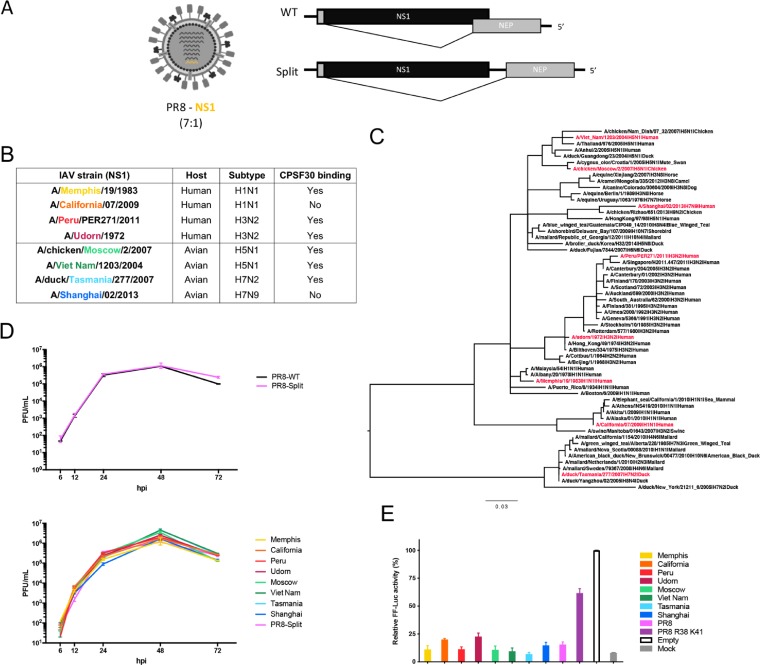
Generation and selection of PR8 recombinant viruses expressing different NS1 proteins. (A) Schematic representation of PR8 recombinant viruses (7:1) bearing NS1 proteins from different origins (PR8-NS1) and representation of the wild-type (WT) segments of eight of influenza A vRNAs which encode two proteins (NS1 and NEP) and modified segment eight encoding separated reading frames (PR8-Split). (B) Selection of PR8-NS1 viruses of interest. (C) Neighbor joining phylogenetic tree of a selection of 58 NS1 amino acid sequences representative of the global diversity of NS1 proteins from influenza A viruses. Sequences used in this study are shown in red. (D) Multicycle replication kinetics of PR8-Split and PR8-WT or PR8-Split and PR8-NS1 viruses in A549 cells. Cells were infected at an MOI of 0.1 with the indicated virus, and supernatants were harvested at various time points for plaque assay titration. Data points show mean values (*n* = 3), and error bars represent standard deviations. (E) Impact of hNS1 and aNS1 proteins on IFN-β induction in human cells. 293T cells were cotransfected with pDZ plasmid encoding the indicated NS1 protein (or empty vector), together with a firefly luciferase (FF-Luc) IFN-β promoter reporter plasmid (p125Luc) and a herpes simplex virus-thymidine kinase promoter-driven *Renilla* luciferase (Ren-Luc) plasmid. At 24 h posttransfection, cells were infected with a DI-rich SeV preparation for 16 h. Relative FF-Luc activity was normalized to the level of the empty vector plus SeV (set to 100%).

To assess the impact of the expression of the NS1 from different strains on the innate immune responses induced by IAV in human DCs, we used a panel of recombinant viruses expressing different NS1 proteins in the same backbone (A/Puerto Rico/08/34, or PR8) (48). Recombinant viruses were engineered to express the NS1 and nuclear export protein (NEP) from two different, nonoverlapping open reading frames (ORFs) (Fig. 1A) to ensure that there were no changes in NEP (see the Materials and Methods section). The criteria for the selection of the NS1 proteins were the following: (i) host (human or avian), (ii) subtype of IAV (H1N1, H3N2, H5N1, H7N9, or H7N2), (iii) ability to interact with CPSF30, therefore inhibiting mRNA maturation of host RNA, and (iv) allele A or B (Fig. 1B). To ensure the appropriate representation of the diversity of NS1 proteins from different subtypes and species in this panel, we performed a phylogenetic analysis including a total of 58 strains (Fig. 1C; selected NS1 proteins are highlighted in red).

Next, we evaluated the replication kinetics of our NS1 recombinant viruses in susceptible human A549 lung epithelial cells (Fig. 1D). First, we found similar replication levels between wild-type PR8 (PR8-WT) and PR8 with the NS1 segment modified (PR8-Split), as shown in Fig. 1D, and when we compared the eight recombinant PR8 viruses expressing NS1 proteins from other selected viruses (PR8-NS1) in A549 cells, we also found very similar profiles of replication in A549 cells among them. These results suggest that viruses expressing the different NS1 proteins replicate efficiently in this human epithelial cell line.

Then, we analyzed the ability of these NS1 proteins to suppress the induction of IFNs in human cells. Human 293T cells were cotransfected with an IFN-β promoter-dependent firefly luciferase (FF-Luc) reporter construct and a constitutively active *Renilla* luciferase (Ren-Luc) expression plasmid, together with plasmids expressing the NS1 proteins of interest (or an empty vector as a control). As shown in Fig. 1E, after infection with a defective interfering (DI) genome-rich Sendai virus (SeV) preparation, human 293T cells induced robust amounts of IFN-β promoter-driven FF-Luc activity when we transfected an empty vector or upon expression of a double mutant of NS1 from PR8 (PR8 R38A K41A) that was previously found to abrogate the RNA binding activity (49, 50). In contrast, IFN-β promoter activity in 293T cells expressing our selected NS1 proteins was strongly repressed, confirming that all the selected NS1 proteins are functional as they are able to efficiently inhibit the induction of type I IFNs.

### Impact of different IAV NS1 proteins on the innate immune response in primary human DCs.

**FIG 2:**
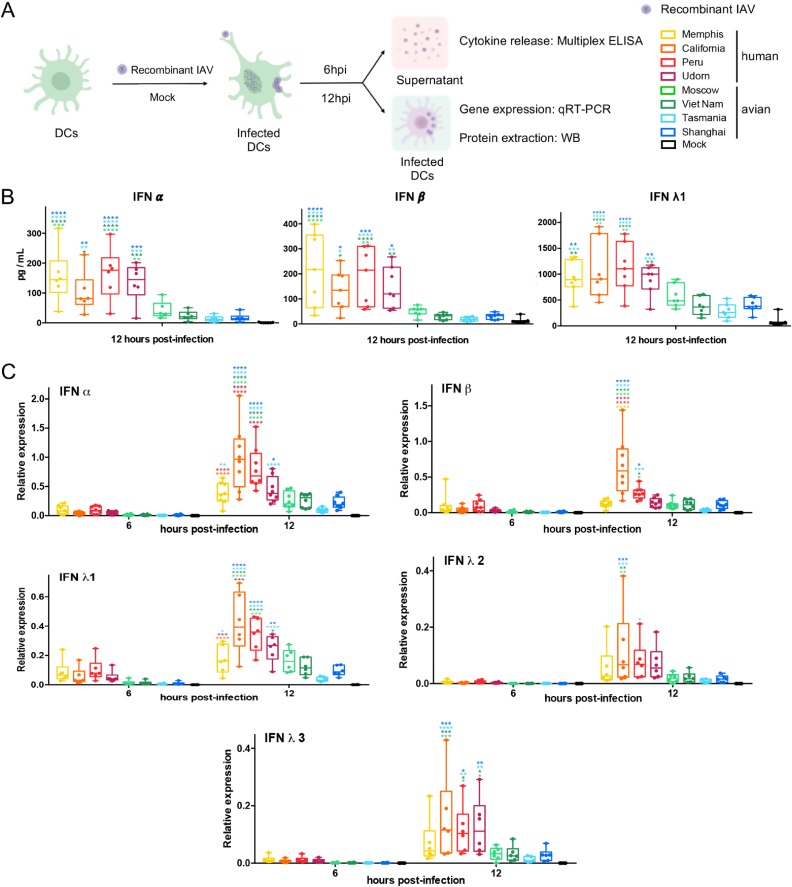
Differential IFN responses induced by recombinant viruses expressing NS1 proteins from human or avian IAVs in primary human DCs. (A) Experimental design created with BioRender. WB, Western blotting. (B) Levels of type I IFN (IFN-α and IFN-β) and type III IFN (IFN-λ1) protein detected in supernatants of infected cells by multiplex ELISA at 12 hpi. (C) Analysis of the expression levels of mRNAs for type I IFN (IFN-α and IFN-β) and type III IFN (IFN-λ1, IFN-λ2, and IFN-λ3) at 6 and 12 hpi, normalized to the level of the housekeeping gene (RPS11) by qRT-PCR. DCs were infected at an MOI of 1 independently with 8 different recombinant viruses. Data from 6 to 8 infected donors are shown. Statistical significance was determined using two-way ANOVA followed by a Tukey’s test for multiple comparisons. Adjusted *P* values are indicated as follows: ****, <0.0001; ***, <0.001; **, <0.01; *, <0.1.

We along with others have previously shown that IAV can infect human DCs and that the NS1 can counteract the innate immune response in those cells (43). As mentioned above, not all NS1 proteins have the same functions present. Therefore, we hypothesized that infection with IAV expressing NS1 proteins from different strains might result in distinct innate immune profiles in human DCs. DCs were infected with the different recombinant viruses (Fig. 1B) at a multiplicity of infection (MOI) of 1. At 6 and 12 h postinfection (hpi), supernatants and cells were collected to analyze their innate immune profile by multiplex enzyme-linked immunosorbent assay (ELISA) and quantitative reverse transcription-PCR (qRT-PCR) (Fig. 2A). As shown in Fig. 2B, DCs infected by recombinant viruses with human NS1 (hNS1; H1N1 and H3N2 isolates) showed higher levels of type I (IFN-α and IFN-β) and type III (IFN-λ1) IFNs than those expressing avian NS1 proteins (aNS1; H5N1, H7N9, and H7N2 isolates) in the supernatants of infected cells at 12 hpi. Similar results were observed when we analyzed the expression of type I and III IFNs by qRT-PCR (Fig. 2C). Interestingly, A/California/07/2009 virus induced higher levels of IFN-α, IFN-β, and IFN-λ1 to IFN-λ3 than the rest of the human NS1-bearing viruses, as determined by qRT-PCR at 12 hpi, a finding which might be associated with the lack of the CPSF30 binding function. However, these differences were not detected at the protein level in the supernatants. Among the different IFNs measured in these experiments, we found that IFN-λ1 showed the most robust expression in DCs.

**FIG 3:**
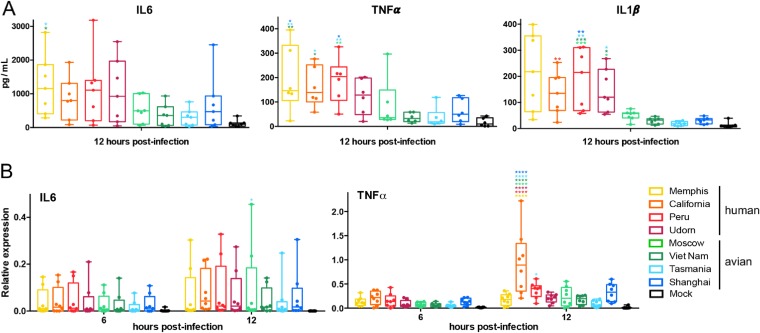
Similar proinflammatory responses induced by recombinant viruses expressing NS1 proteins from human or avian IAVs in primary human DCs. (A) Levels of IL-6, TNF-α, and IL-1β protein detected in supernatants of infected cells by multiplex ELISA at 12 hpi. (B) Analysis of the expression of mRNA levels for IL-6 and TNF-α at 6 and 12 hpi normalized to the level of RPS11 by qRT-PCR. DCs were infected at an MOI of 1 independently with 8 different recombinant viruses. Data from 7 to 8 different infected donors are shown. Statistical significance was determined using two-way ANOVA, followed by Tukey’s test for multiple comparisons. Adjusted *P* values are indicated as follows: ****, <0.0001; ***, <0.001; **, <0.01; *, <0.1.

We also analyzed the production of interleukin-6 (IL-6), tumor necrosis factor alpha (TNF-α), and IL-1β, three proinflammatory cytokines associated with DC activation under NF-κB transcription factor regulation (1, 51, 52). Similar levels of IL-6 were detected in the supernatant of DCs infected with the different viruses as determined by multiplex ELISA at 12 hpi (Fig. 3A). In the case of TNF-α and IL-1β, we observed that cells infected with hNS1-expressing viruses tended to produce higher levels than those infected by aNS1-expressing viruses. However, this differential pattern between hNS1 and aNS1 was not detected at the mRNA level by qRT-PCR at either 6 or 12 hpi (Fig. 3B). Similarly to what we observed for IFN expression, A/California/07/2009 virus also showed significantly higher levels of TNF-α expression by 12 hpi than the rest of the viruses.

Altogether, these data indicate that the host origin of the NS1 protein has a clear impact on the induction of the expression of type I and type III IFNs by infected DCs, where viruses expressing hNS1 induce a stronger global type I and III response than those expressing aNS1. Also, we found that IFN-λ1 was more highly released into the supernatants than IFN-α or IFN-β, highlighting a role for this cytokine in the innate immune response to IAV infections. Slightly stronger proinflammatory responses were found in cells infected by hNS1-bearing strains, indicating some differential effect between hNS1 and aNS1 also in NF-κB-mediated induction of cytokines.

### Effect of different IAV NS1 proteins on virus replication and expression of viral proteins in primary human DCs.

**FIG 4:**
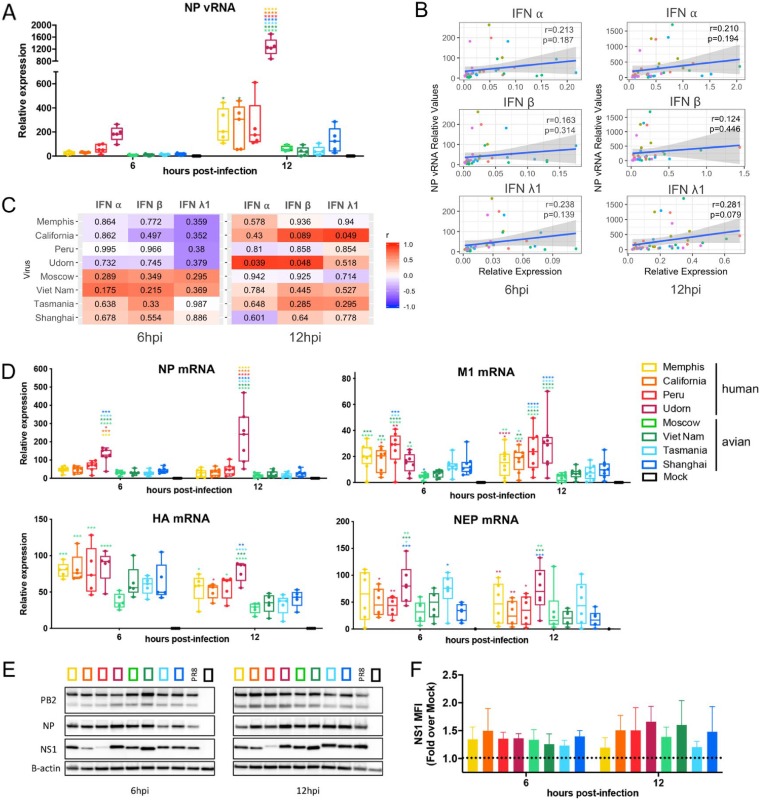
Characterization of recombinant IAVs expressing NS1 proteins from human or avian viruses in primary human DCs. (A) Expression of vRNA levels for NP at 6 and 12 hpi by qRT-PCR. (B) Analysis of the correlation between levels of intracellular vRNA and IFN induction across all the samples from 5 donors (individual viruses are indicated with differently colored data points). The blue line indicates the linear regression model fit to the observed data, and the shadow indicates the 95% confidence interval for predictions from that linear model. Pearson’s coefficient (*r*) and the *P* value for the correlation (*P*) are shown in each plot. (C) Heat maps showing the correlation between vRNA levels and IFN induction for samples from different infected donors. Color scale indicates the Pearson’s correlation coefficient (*r*), and labels in the heat map cells indicate the *P* value for that correlation. (D) Analysis of the expression of mRNA levels for NP, M1, HA, and NEP at 6 and 12 hpi by qRT-PCR. (E) Expression of viral PB2, NP, and NS1 protein levels and β-actin in infected DCs at 6 and 12 hpi by Western blotting of samples from one representative donor of three independent experiments with different donors. (F) MFIs for NS1 proteins in infected DCs at 6 and 12 hpi are shown. Bars represent means ± standard deviations of four different donors. Statistical significance (A, D, and F) was determined using two-way ANOVA followed by Tukey’s test for multiple comparisons. Adjusted *P* values are as indicated follows: ****, <0.0001; ***, <0.001; **, <0.01; *, <0.1.

During infection, viral RNA detection by sensors such as RIG-I or Toll-like receptor 3 (TLR3) results in the induction of type I or type III IFNs as well as of proinflammatory cytokines. Therefore, to study whether the differences in the induction of the IFN response are explained by differences in levels of intracellular replication, we measured the levels of nucleoprotein (NP) viral RNA (vRNA) by qRT-PCR specifically for the negative RNA strand of the segment in DCs after infection with our panel of recombinant viruses. As shown in Fig. 4A, no significant differences were found at 6 hpi among the different viruses. At 12 hpi, A/Udorn/1972 showed statistically significant higher levels of replication than the rest of the viruses. Despite the fact that the differences among the remaining seven viruses are not statistically significant, viruses with hNS1 tended to show slightly higher levels of intracellular replication than viruses with aNS1. For a better understanding of the relationship between intracellular viral replication and IFN induction, we performed a correlation analysis of these variables. A low level of correlation was observed globally between vRNA levels and IFN-α, IFN-β, or IFN-λ1, as indicated by the Pearson’s correlation coefficients (*r*) (Fig. 4B) (none of the *P* values indicated a significant association). The percentages of the variation in IFN induction explained by the intracellular replication (estimated by *r*^2^ values) were around 2% and 8%, indicating that other factors, in addition to some small contribution of the replication of the virus, might be responsible for the differences in the innate immune responses induced by viruses bearing hNS1 or aNS1. We also studied the vRNA-IFN correlation in detail for the separate viruses (Fig. 4C). Using this method, we still did not find statistically significant correlations, with the exception of A/California/07/2009 for IFN-λ1 or A/Udorn/1972 for IFN-α and IFN-β at 12 hpi. Remarkably, at 6 h we found an interesting and distinct negative trend between vRNA and all of the IFNs for hNS1-bearing viruses, while a positive trend was observed for aNS1-expressing viruses, suggesting that hNS1 and aNS1 could be affecting this relationship differently.

In order to investigate the effect of the different NS1 proteins on the expression of other viral proteins in DCs, we analyzed the levels of expression of multiple viral genes by qRT-PCR and Western blotting. Analysis of the levels of NP mRNA showed similar results across all the viruses, except for A/Udorn/1972, which showed higher levels of vRNA NP expression in infected DCs than the rest of the viruses (Fig. 4A). Interestingly, similar levels of NP protein and PB2 were found in cells infected with all viruses by Western blotting (Fig. 4E). In the case of M1 mRNA, we found a different pattern between hNS1- and aNS1-bearing viruses, suggesting that the presence of different NS1 proteins in the cell could affect the expression of this gene (Fig. 4D). We also analyzed the expression of hemagglutinin (HA) and NEP mRNAs in cells infected by the different viruses (Fig. 4D). In this case, a similar trend to the one we observed for M1 was found although differences across viruses were not significant in most cases. Surprisingly, A/Udorn/1972 did not show higher expression of M1, HA, or NEP in infected cells than the rest of the viruses, as observed for NP mRNA. In addition, we also analyzed the expression of NS1 protein by Western blotting to confirm that this protein is expressed during infection in DCs by all of the viruses tested. We found slight differences in NS1 expression levels across the samples infected with our NS1 recombinant viruses as measured by Western blotting. However, we did not find a different pattern between cells infected with viruses expressing hNS1 and those expressing aNS1 proteins. Since the sequences of all of the NS1 proteins are notably different (see Fig. 8B), the variations in the expression levels that we observed could be due to differences in the levels of stability of the protein itself or to differences in affinity to the NS1 antibody (polyclonal antibody 1-73 [53]). In order to make sure that NS1 levels of expression were similar, we have also measured the NS1 mean fluorescence intensity (MFI) in infected DCs from four different donors by imaging flow cytometry at 6 and 12 h postinfection (Fig. 4F). Results showed similar MFIs (fold change in expression over mock infection level) for each NS1, and the analysis of variance (ANOVA) test performed reveled that differences in NS1 expression levels across the different viruses in infected DCs were not statistically significant.

Overall, we found that the different NS1 proteins expressed by our panel of recombinant viruses had no significant effect on NP vRNA and mRNA expression in infected DCs (except for A/Udorn/1972) or on HA or NEP mRNA or viral protein expression in those infected cells. However, different levels of mRNA for M1 were found between hNS1- and aNS1-expressing viruses, similar to the profile that we identified for the induction of IFN responses.

### Impact of different IAV NS1 proteins on the innate immune response in primary NHBE cells.

To study the innate immune responses induced by our panel of recombinant viruses and their replication in respiratory epithelial cells, we used differentiated normal human bronchial epithelial (NHBE) cell cultures. These cells form a polarized pseudostratified epithelium with different epithelial cell types when cultured in an air-liquid interface, which recapitulates the respiratory physiological environment (54).

**FIG 5:**
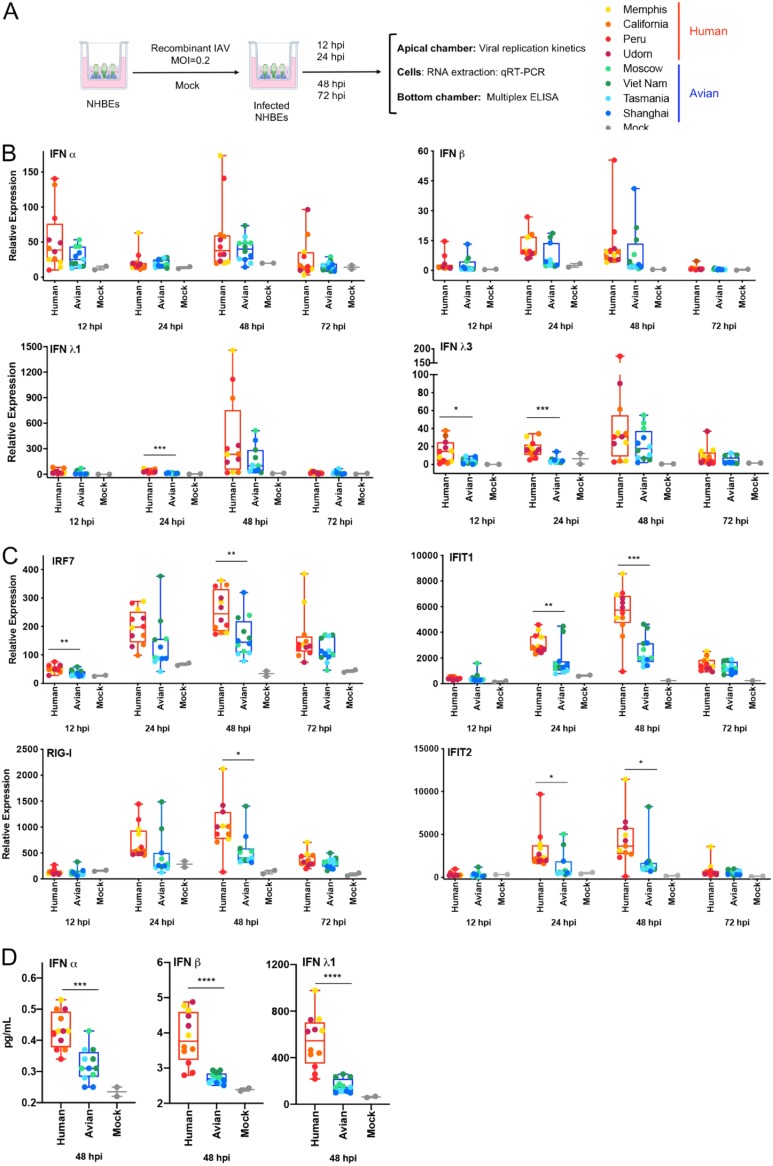
Differential immune responses induced by recombinant viruses expressing NS1 proteins from human or avian IAVs in primary NHBE cells. (A) Experimental design created with Biorender. (B) Analysis of the expression of mRNA levels for type I IFN (IFN-α and IFN-β) and type III IFN (IFN-λ1 and IFN-λ3). (C) ISG IRF7 and IFIT1 levels at different time points by qRT-PCR. (D) Levels of type I IFN (IFN-α and IFN-β) and type III IFN (IFN-λ1) protein detected in bottom chambers of infected cells by multiplex ELISA at 48 hpi. Data were grouped as human or avian depending the origin of the NS1 proteins carried by the viruses. Data from 3 biological replicates for each virus are shown (dots); each column represents the mean of the group with the corresponding standard error of the mean. Statistical significance was determined using a *t* test. *P* values are as indicated follows: ****, <0.0001; ***. <0.001; **, <0.01; *, <0.1.

We infected the apical surface of the NHBE cells with our eight different IAV recombinant viruses at an MOI of 0.2. At 12, 24, 48 and 72 hpi, samples were collected to analyze the innate immune profile (by qRT-PCR in cell lysates and cytokine release to the medium in the bottom chamber by multiplex ELISA) and the viral replication kinetics (washes from the apical chamber by plaque assay) (Fig. 5A).

As shown in Fig. 5B, NHBE cells infected with recombinant viruses expressing hNS1 showed higher expression levels of type I and III IFNs as measured by qRT-PCR than those bearing aNS1 proteins. However, these differences were statistically significant only for IFN- λ1 and IFN-λ3 at early time points (12 and/or 24 hpi). Importantly, it is known that type III IFNs play an essential role in the innate immune defense in epithelial cells during IAV infection (6, 7, 55). IFNs are secreted molecules that interact with specific cellular receptors inducing the expression of IFN-stimulated genes (ISGs). Several of these ISGs have antiviral and immune-modulatory properties and therefore have an important impact on the control of the spread of IAV in the respiratory epithelium. Consequently, we measured the expression of several ISGs by qRT-PCR. We found that the expression levels of IRF-7, IFIT1, IFIT2, and RIG-I (Fig. 5C) showed statistically significant differences after infection with hNS1-expressing recombinant viruses or aNS1-expressing recombinant viruses at certain time points. Interestingly, these data reflected the dynamics of the IFN-mediated stimulation of these genes since in all cases the greater differences were found at later time points than in the case of IFN-λ1 and IFN-λ3 (24 to 48 hpi versus 12 to 24 hpi). We confirmed the differences observed in IFN production after infection with aNS1- and hNS1-expressing IAVs by multiplex ELISA at 48 hpi (Fig. 5D). In this case, the differences for IFN-α and IFN-β expression levels were also statistically significant. Of note, the levels of IFN-α and IFN-β were strikingly lower than those for IFN-λ1. In this assay, accumulation of protein over 48 h is measured, which could result in increased sensitivity compared to that of a qRT-PCR assay.

**FIG 6:**
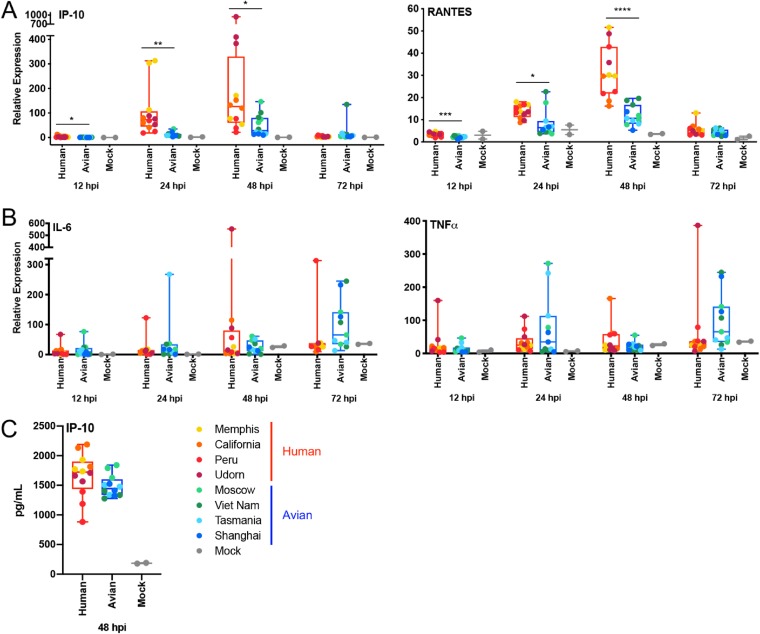
Chemokines and proinflammatory responses induced by recombinant viruses expressing NS1 proteins from human or avian IAVs in primary NHBE cells. (A and B) Analysis of the mRNA expression levels for chemokines IP-10 and RANTES and proinflammatory cytokines IL-6 and TNF-α, as indicated, at different time points, normalized to the level of a housekeeping gene by qRT-PCR. (C) Levels of IP-10 protein detected in bottom chambers of infected NHBE cells by multiplex ELISA at 48 hpi. Data were grouped as human or avian depending the origin of the NS1 protein carried by the virus. Data from 3 biological replicates for each virus are shown (dots); each column represents the mean of the group with the corresponding standard error of the means. Statistical significance was determined using the *t* test. *P* values are indicated as follows: ****, <0.0001; ***, <0.001; **, <0.01; *, <0.1.

We also analyzed by qRT-PCR the expression of two chemokines, IP-10 and RANTES (Fig. 6A), which showed a similar expression pattern, with increased levels in cells infected with viruses expressing hNS1 over levels in those expressing aNS1. This pattern was also observed in the levels of IP-10 measured by multiplex ELISA at 48 hpi. In line with the data in DCs, for the proinflammatory cytokines IL-6 and TNF-α, levels of expression were independent of the virus used to infect the NHBE cells (Fig. 6B).

**FIG 7:**
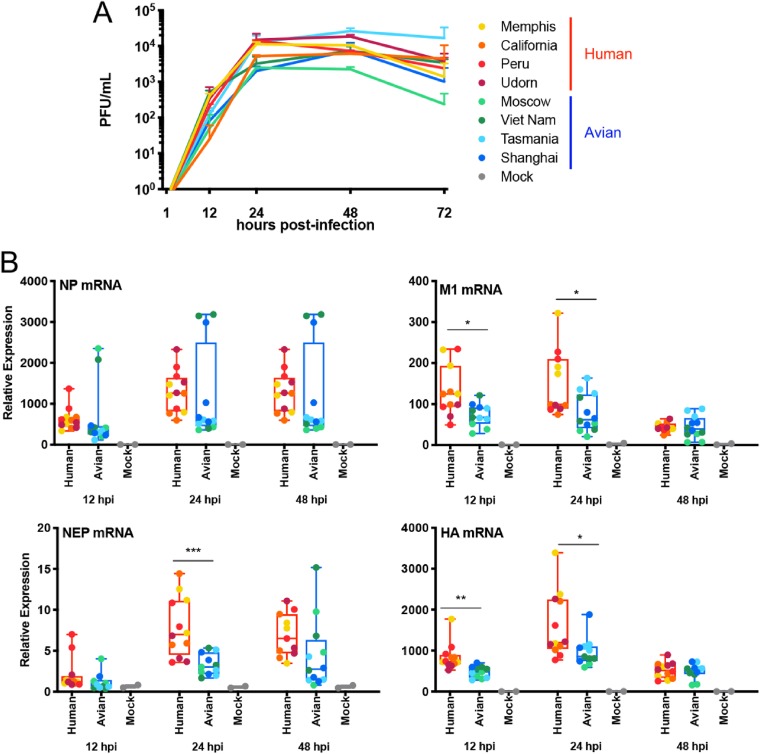
Characterization of recombinant IAVs expressing NS1 proteins from human or avian viruses in primary NHBE cells. (A) Analysis of multicycle replication kinetics of the recombinant viruses in NHBE cells infected at an MOI of 0.2 with the indicated virus as measured by plaque assay. Data points show mean values of 3 biological replicates, and error bars represent standard errors of the means. (B) Expression levels of mRNAs for NP, M1, NEP, and HA in infected NHBE cells at different time points by qRT-PCR. Data were grouped as human or avian depending the origin of the NS1 proteins carried by the viruses. Each column represents the mean of the group (*n* = 3) with the corresponding standard error of the means. Statistical significance was determined using a *t* test. *P* values are indicated as follows: ****, <0.001; ***, <0.001; **, <0.01; *, <0.1.

NHBE cells produce human airway trypsin-like (HAT) protease that allows IAV multicycle replication, with the consequent release of new viral particles from the apical side of the cells (56). As shown in Fig. 7A, all viruses have the ability to perform multicycle replication in NHBE cells, but no major differences were observed across viruses.

Next, we analyzed the levels of expression of multiple viral genes by qRT-PCR, and the differential patterns (human versus avian) previously observed in infected DCs were also observed in infected NHBE cells. NHBE cells infected with viruses bearing hNS1 proteins showed higher levels of expression of NP, M1, HA, and NEP mRNAs than NHBE cells infected with viruses bearing aNS1 proteins (Fig. 7B), and some of these differences were statistically significant by 12 and/or 24 hpi.

Altogether, we found that recombinant IAVs expressing NS1 proteins from human or avian IAV origin induced a differential immune response in both DCs and in NHBE cells. The immune response was characterized by higher levels of expression of IFNs and ISGs in cells infected with hNS1-bearing viruses than in aNS1-bearing viruses. In addition, in infected NHBE cells, higher levels of viral mRNAs were detected after infection with hNS1-bearing viruses than with aNS1-bearing viruses although these differences did not result in increased viral particle release.

### hNS1 and aNS1 have different dynamics of nuclear localization during primary human DC infection.

**FIG 8:**
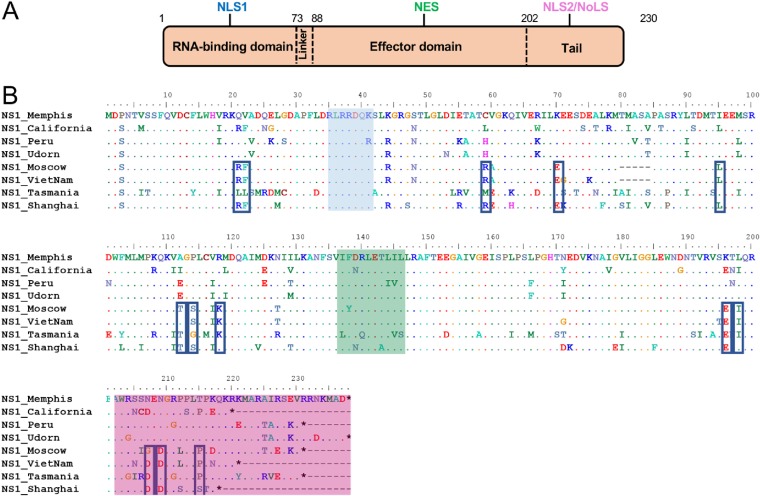
Multiple sequence alignment of full-length NS1 proteins from IAVs. (A) Schematic representation of an NS1 monomer with the two functionally different domains, the N-terminal RNA binding domain and the C-terminal the effector domain, separated by a short linker region. The last residues of the C terminus form a tail. The NS1 protein is 218 to 237 amino acids long depending upon the strain. (B) Amino acid sequence alignment of the NS1 proteins from IAVs included in this study: A/Memphis/19/1983 (H1N1), A/California/07/2009 (H1N1), A/Peru271/2011 (H3N2), A/Udorn/1972 (H3N2), A/chicken/Moscow/2/2007 (H5N1), A/Vietnam/277/2007 (H5N1), A/duck/Tasmania/277/2007 (H7N2), and A/Shanghai/02/2003 (H7N9). Identity (·) and amino acid changes between strains are indicated. Blue shading highlights NLS1, green shading indicates the NES, and pink shading indicates NLS2. Black boxes indicate residues that are different between human and avian proteins and common in at least 3 out of 4 sequences.

**FIG 9:**
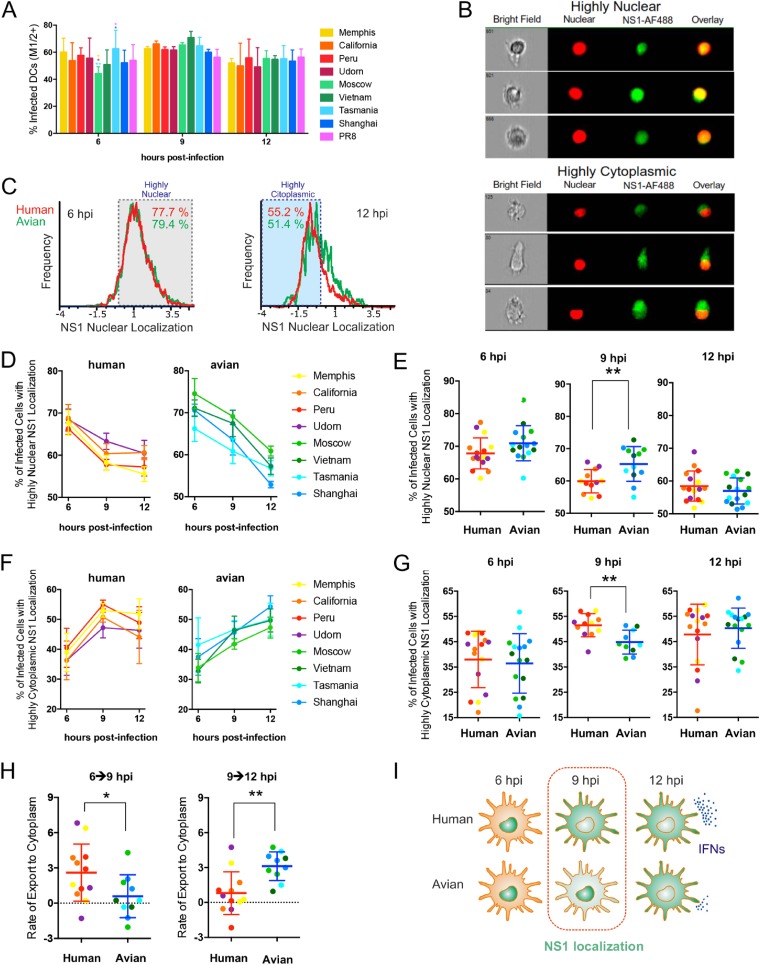
hNS1 and aNS1 have different kinetics of nuclear export during primary human DC infection. (A) Infectivity in DCs measured as the percentage of M1/2-positive cells (MOI of 1 PFU/cell). Cells were stained, fixed, permeabilized, and stained with M1/2 E10-PacBlue, NS1-AF488, and a nuclear marker (DRAQ5). Images were obtained using an ImageStream instrument (Amnis). In order to quantify the localization of NS1-AF488 in the nucleus for each cell, a bright detail similarity (BDS) score was calculated between the images (NS1 nuclear localization score; see Materials and Methods). (B) Representative images of cells in which NS1 is localized mainly in the nucleus (highly nuclear) or in the cytoplasm (highly cytoplasmic). (C) Representative histograms. (D to G) Percentage of infected cells with highly nuclear or cytoplasmic localization per virus (D and F) or per time point (E and G), grouped by avian or human strains. (H) Rate of export to the cytoplasm (percent change per hour) for human and avian strains from 6 to 9 hpi and for 9 to 12 hpi. *, *P* < 0.05; **, *P* < 0.01. (I) Working model. NS1 localization (in green) during time.

To identify sequence variations that could account for the different innate immune profiles induced by viruses expressing hNS1 or aNS1, we performed an alignment of the eight NS1 amino acid sequences and searched for amino acids differentially conserved between both groups (Fig. 8B). Specifically, we considered residues (i) that are conserved in at least three of the four NS1 proteins within each group and (ii) that were different between the two groups. We observed that in the C-terminal region there were few positions (positions 196, 198, 207, 209, and 215) with striking differences between aNS1 and hNS1, which could affect the nuclear localization sequence NLS2. Because NS1 exerts multiple functions in both the nucleus and cytoplasm, the cellular localization of this protein could have important consequences during infection. Previous studies have shown that NS1 is early localized predominantly in the nucleus and that it is translocated into the cytoplasm at late stages of infection. These intracellular localization patterns vary depending on virus strain, expression levels of NS1, cell type, cell polarity, and time postinfection (42, 57). Therefore, we next hypothesized that hNS1 and aNS1 could have different nuclear localization dynamics, which could be associated with the differential innate immune responses induced during infection. To address this, we analyzed the localization dynamics of NS1 for our eight different NS1-expressing recombinant viruses during infection in DCs using imaging flow cytometry (ImageStream Mark II; Amnis). Cells were infected during 6, 9, and 12 h, fixed, and stained for NS1 and M1/M2 proteins and with DRAQ5 for nuclear staining. Infected cells were selected for subsequent analysis by gating in M1/M2-expressing cells (Fig. 9A). Then, a nuclear localization score for NS1 was calculated based on the colocalization of NS1 expression and nuclear signal (Fig. 9B). Based on this score, we determined the percentage of cells with a predominantly nuclear signal (highly nuclear) and the percentage of cells with a predominantly cytoplasmic signal (highly cytoplasmic) (Fig. 9C) for samples infected with each of the viruses. As expected, we found a decrease in the nuclear localization over time for all of the NS1 proteins. However, the dynamics of this reduction in nuclear localization were clearly different between hNS1 and aNS1 (Fig. 9D). hNS1 localization was readily reduced at 9 h, while the decay in the case of aNS1 was more pronounced from 9 to 12 hpi. When we grouped hNS1 and aNS1 for comparison, we found a significantly higher number of cells with nuclear localization in aNS1 by 9 hpi than in hNS1 (Fig. 9E) as a consequence of their different dynamics. We also evaluated the percentage of cells with higher cytoplasmic localization. Similarly, these data indicated that hNS1 was translocated to the cytoplasm earlier than aNS1 (Fig. 9F), which resulted in a higher number of cells expressing NS1 in the cytoplasm by 9 hpi (Fig. 9G). Consistently, when we compared the rate of export to the cytoplasm during the two different time intervals, we found significantly faster and earlier kinetics for hNS1 from 6 hpi to 9 hpi, while aNS1 showed a faster decrease in the following time period, from 9 to 12 hpi (Fig. 9H).

In summary, we found that the different NS1 proteins expressed in the same backbone virus (PR8) present clear differences in their dynamics of cellular localization in infected DCs depending on their host origin. Specifically, NS1 proteins from human IAVs are mobilized faster from the nucleus to the cytoplasm than NS1 proteins from avian viruses. Given that NS1 has multiple and different functions in the nucleus and in the cytoplasm, the different immune response profiles found between hNS1 and aNS1 are likely associated with these distinct localization pattern kinetics (Fig. 9I).

## DISCUSSION

The IAV NS1 protein is a multifunctional protein capable of inhibiting innate immune responses to IAV infection, and this inhibition is needed in order for the virus to successfully spread in the infected organs and eventually get transmitted to new hosts (14). Several studies have identified the NS1 protein as one of the determinants associated with highly virulent features of some influenza A viruses in mammalian hosts (58–60). As NS1 proteins are highly variable among different strains, we hypothesized that there is a strain-specific effect of NS1 in the modulation of the innate immune responses in human primary cells during IAV infection. Human infections by avian IAV often result in severe disease, characterized by high levels of cytokines and increased immunopathology (reviewed in reference 61). The high virulence of avian IAV in humans involves multiple factors, including the NS1 protein (reviewed in reference 43). Interestingly, we found that infection of human DCs and NHBE cells with viruses expressing NS1 from human or avian IAV results in different patterns of innate immune responses. These data suggest that avian influenza viruses might be more efficient at antagonizing innate immune responses in human primary cells since infection with aNS1-bearing viruses induced lower levels of type I and III IFNs than infection with hNS1-bearing viruses, supporting the hypothesis that intrinsic characteristics of the avian NS1 proteins contribute to the increased virulence of avian IAV in humans.

IFNs are widely expressed cytokines that act as the first line of defense against viral infections and possess the ability to induce an antiviral state in the producing as well as neighboring cells. Even though the signal transduction cascades are similar between type I and type III IFNs (7), it has been reported that type III IFNs precede a type I IFN antiviral defense in the respiratory tract in mice (6). Our results in infected DCs and NHBE cells also show that the amount of IFN-λ1 detected in the supernatant is higher than the amount of IFN-α/β (also observed in mRNA levels), in agreement with the idea that type III IFNs are the first IFNs produced to suppress the initial viral spread without activating systemic inflammation (6).

In search of possible mechanisms for the differences in the innate immune responses between IAVs bearing hNS1 or aNS1, we analyzed their intracellular replication profiles (measuring vRNA) and expression of viral proteins (measuring mRNA and protein) in infected cells. In general, we found that in infected DCs differential levels of replication or protein expression did not correlate with the innate immune patterns elicited by viruses with human or avian NS1 proteins. This was also the case for NP mRNA expression. However, there was a specific gene, M1, that seemed to be greatly influenced by the host origin of the NS1 protein in infected DCs and NHBE cells. Expression levels of other genes such as HA and NEP were also altered by the host origin of the NS1 protein, but multicycle replication kinetics were similar between viruses bearing hNS1 and aNS1 in infected NHBE cells.

The alignment of the eight different NS1 proteins used in this study allowed the identification of residues that might be involved in the different innate immune responses. We identified amino acids at the following positions: 21, 22, 59, 70, and 71 in the RNA-binding domain; 95, 112, 114, 118, 127, 196, and 198 in the effector domain; and 207, 209, 212, and 215 in the C terminus or tail. Differences in specific amino acids can have implications during infection. For example, some amino acids can modify the structure of NS1, which could impact the interactions of the NS1 protein with other proteins. In our selected viruses only avian NS1 proteins (with the exception of the allele B) have an R in position 59. It is known that this amino acid is involved together with Q63 of the RNA binding domain and G168 and T170 of the effector domain in the formation of a hydrogen bond that interconnects the two domains, which stabilize the structure (62). In addition to these four amino acids, the residue composition at position 71 (that is also different between hNS1 and aNS1) gives a different degree of flexibility/plasticity to the NS1 dimer, a fact that could influence the interactions of NS1 with some other proteins (62).

Another major function of NS1 is to block activation of protein kinase R (PKR) (26), a critical component of the cellular antiviral system. It has been shown that the inhibition of PKR activation by Udorn NS1 *in vitro* requires a specific amino acid sequence between positions 123 and 127 of the NS1 since mutants with alanine residues at positions 123 and 124 or at positions 126 and 127 fail in this inhibition (26, 63). In our selected NS1 proteins, amino acids at residue 127 are different between hNS1 and aNS1, which could potentially result in differences in their abilities to block PKR activation.

A report by Kuo and collaborators (64) demonstrated that a variation at amino acid position 196 (K or E) of the NS1 protein determines its ability to block the activation of IRF3 and IFN-β transcription. Specifically, the investigators observed that NS1 proteins from H3N2, H2N2, and some H1N1 IAVs do not block the activation of IRF3 or IFN-β transcription and that all contained a K196 residue, whereas the NS1 proteins from H5N1 and some H1N1 viruses, that are able to block these activations, contained E196 instead. Our results concur with this observation since H1N1 (with the exception of A/California/07/2009) and H3N2 viruses bearing hNS1 have K at position 196 and induce higher levels of expression of IFN mRNAs during DC infection than H5N1, H7N2, and H7N9 aNS1-bearing viruses, which have an E at position 196 (Fig. 2C).

Additionally, NS1 can be regulated by posttranslational modifications such as phosphorylation, SUMOylation, or ISGylation (9). We found that residue K70 is present in human NS1 proteins and that it can be SUMOylated (65), in contrast to avian NS1 proteins, which present E in the same position. Interestingly, alterations in NS1 SUMOylation have been shown to affect the ability of NS1 to inhibit the IFN response (65); therefore, this amino acid difference could also contribute to the phenotype observed in DCs upon infection with aNS1- and hNS1-bearing viruses.

Since it has been suggested that subcellular trafficking of NS1 is a determinant of host adaptation (66), we also evaluated the cellular localization dynamics for these different NS1 proteins in human DCs during the infection. We found that viruses bearing hNS1 have different localization kinetics from viruses with aNS1. Specifically, hNS1 exits from the nucleus at a higher rate than aNS1. Multiple NS1 functions occur in the nucleus and cytoplasm during influenza infection (41, 57, 67); therefore, its intracellular localization dynamics need to be highly regulated (14). Given these results, we propose that there is an association between the differential immune phenotype observed in human DCs, the intracellular localization dynamics of NS1, and the expression of M1. Previous studies have reported that NS1 can bind M1 mRNA and regulate M1 mRNA splicing (68) and also that this process occurs through nuclear speckles (69).

NS1 proteins contain two nuclear localization signals (NLS), NLS1 and NLS2, and one nuclear export signal (NES) to regulate their nuclear trafficking of IAV (Fig. 9). Despite the variability of the NS1 protein sequences, NLS1 is very conserved across different strains and is located in the RNA binding domain involving amino acids 34 to 38 (DRLRR) (41). On the other hand, NLS2 is a very variable region located in the C-terminal domain of the protein. NLS2 was initially mapped between amino acids 203 and 237 (41), with a putative nuclear localization motif between residues 216 and 221 (P-X-Q-K-R-K/E) (70). It has been proposed that NLS interacts with the cellular protein importin α (67) and induces rapid nuclear localization after its translation (71). Also, it is thought that other amino acids less proximal to the C terminus might also be involved in the NLS2 signature (72) since altering the NLS2 sequence for several human strains does not seem to have an effect on NS1 nuclear localization, while for avian viruses the presence of both (in mammalian cells) is required. In addition to that, a nucleolar localization signal (NoLS) also has been described for some strains. NoLS is associated with basic amino acids at positions 219, 220, 224, and 229 and also at positions 231 and 232 in NS1 proteins with C-terminal extensions (67, 73). However, the role of the nucleolar localization for NS1 and its contribution to viral replication are unknown. Finally, the NES is a quite conserved region, located in the effector domain, specifically, amino acids F138, L141, L144, and L146 (42). In addition, a previous study found that the region between amino acids 100 and 120, in which we found a small cluster of different amino acids, might also be involved in nuclear export (74). However, no major sequence differences were observed in NLS1 and NES regions between the NS1 sequences used in our study. In contrast, we found multiple differences between hNS1 and aNS1 in their NLS2 sequences and surrounding domains, which could be associated with their localization phenotypes. Additionally, we cannot exclude the possibility of other currently undescribed amino acids that could be responsible for this phenotype. Specific interactions between cellular and viral factors with NS1 proteins play key roles in determining the intracellular localization of NS1 by masking or unmasking specific subcellular localization signals (42). Therefore, the different localization dynamics of human and avian NS1 proteins could result from specific interaction partners for hNS1 proteins in the nucleus that unmask the NES and allow faster transport to the cytoplasmic compartment than that of aNS1 proteins. Alternatively, aNS1 proteins may interact with specific proteins in the nucleus that retain them longer in the nucleus than hNS1 proteins. Further studies of the interactions between NS1 proteins and different proteins in the nucleus will help elucidate the mechanism behind the different localization dynamics.

Altogether, our results showed differential immune profiles in both human DCs and NHBE cells after infection with recombinant IAV viruses expressing the NS1 from avian or human viruses. These different phenotypes are characterized by higher expression of type I and type III IFNs in human DCs and NHBE cells and also by higher expression of ISGs in NHBE cells infected with viruses bearing human NS1 than with viruses bearing avian NS1. Remarkably, this phenotype is not due to differences in intracellular viral replication (DCs) or in viral particle release (NHBE cells). In addition, differences in the expression levels of some viral genes and in the cellular localization dynamics were found between human and avian NS1 proteins during infection in DCs, which might be associated with the immune phenotype of DCs upon infection with these viruses. Multiple amino acid changes are present between NS1 proteins of IAVs from human and avian origin, and some of them have been previously reported to participate in the immune-modulatory capabilities of NS1. Therefore, the phenotypic differences found between viruses expressing these NS1 proteins are likely a result of the combination of multiple factors. Further research will help to improve our understanding on the IAV NS1 functions at a global level.

## MATERIALS AND METHODS

### Cells.

Human primary DCs were generated from CD14^+^ cells isolated from buffy coats of healthy human donors (New York Blood Center) as described previously (44). Briefly, peripheral blood mononuclear cells (PBMCs) were obtained by Ficoll density gradient centrifugation (Histopaque; Sigma-Aldrich), and CD14^+^ cells were purified using CD14 antibody-labeled magnetic beads and iron-based MiniMACS liquid separation columns (Miltenyi Biotech). CD14^+^ cells were cultured at a final concentration of 10^6^ cells/ml for 5 days at 37°C in RPMI 1640 medium containing 10% fetal bovine serum (FBS) (HyClone; Thermo Fisher Scientific), 2 mM l-glutamine, 1 mM sodium pyruvate, 100 U/ml penicillin, and 100 mg/ml streptomycin (Gibco, Invitrogen) (Complete DC medium) and were supplemented with 500 U/ml human granulocyte-macrophage colony-stimulating factor (hGM-CSF) and 1,000 U/ml human interleukin-4 (hIL-4) (PeproTech).

Madin-Darby canine kidney (MDCK), A549, and 293T cells were obtained from ATCC (Manassas, VA, USA) and were maintained in minimal essential medium (MEM) in the case of MDCKs or in Dulbecco’s modified Eagle medium (DMEM; Gibco, Invitrogen) in the case of 293T and A549 cells, supplemented with 10% FBS, 100 U/ml penicillin, and 100 mg/ml streptomycin (Gibco, Invitrogen).

Primary normal human bronchial epithelial (NHBE) cells were sourced from Lonza (NHBE CC-2540s; Lonza, Walkersville, MD) and cultured in bronchial epithelial growth medium (BEGM; Lonza, Walkersville MD) for an initial propagation. Cells were seeded at ∼500,000 cells/T75 flask and incubated at 37°C in 5% CO_2_. Once cells reached 70 to 80% confluence, they were dissociated and subcultured again by seeding 3,500 cells/cm^2^ into T75 flasks. Once cells had reached 70 to 80% confluence, they were dissociated and seeded on 12-mm Transwells (Corning, Fisher Scientific) coated with 0.3 mg/ml collagen I from rat tail (Corning, Fisher Scientific). NHBE cells were seeded at 100,000 cells/Transwell, and Gray’s medium was used to feed cells during 4 to 5 days or until 100% confluence was reached. When cells reached confluence, the apical medium was removed, and the basal medium was replaced with Gray’s medium supplement with retinoic acid (Sigma) for a final concentration of 15 ng/ml. Medium was changed every second day. NHBE cells were grown for 5 weeks, air lifted (37°C in 5% CO_2_), until they fully differentiated in a polarized pseudostratified epithelium.

### Recombinant viruses.

Recombinant IAVs were generated by reverse genetics techniques as previously described (75). Briefly, these viruses were rescued in the A/Puerto Rico/8/1934 (PR8) background and contain a modified split NS segment where the splice acceptor site of NS1 has been mutated, thus allowing in-frame transcription of both NS1 and NEP transcripts (Fig. 1A). A total of eight recombinant PR8 viruses expressing NS1 proteins from different strains and origins as well as split PR8 were selected from an IAV barcoded library (48): A/Memphis/19/1983 (Memphis), A/California/07/2009 (California), A/Peru271/2011 (Peru), A/Udorn/1972 (Udorn), A/chicken/Moscow/2/2007 (Moscow), A/Vietnam/277/2007 (Vietnam), A/duck/Tasmania/277/2007 (Tasmania), and A/Shanghai/02/2003 (Shanghai) (Fig. 1B).

Viruses were grown in 9-day-old embryonated chicken eggs (SPAFAS; Charles River Laboratories) and were titrated by plaque assay on MDCK cells by following standard procedures. In addition, qRT-PCR was performed with same amount of each virus calculated by the plaque assay titer in order to avoid some misleading titrations. Results from both assays showed equal virus amounts for all preparations.

Viruses were sequenced on Illumina MiSeq platform with a MiSeq reagent kit (version 3; Illumina). Sequence reads were analyzed on Illumina BaseSpace. Alignment of reads to viral genomes was performed using Bowtie and subsequently visualized using the Integrative Genomics Viewer (76).

### Infections of primary human DCs and NHBE cells with the recombinant IAV.

Infectivity of PR8-Split in DCs was analyzed by detecting the proportion of infected cells by flow cytometry at different multiplicities of infection (MOIs) by 24 hpi. An MOI of 1 infected 66% of DCs (data not show), and it was the MOI selected for the innate immune profiling after IAV infection with PR8-NS1.

DCs were infected with the NS1 recombinant IAV at the MOI of 1 using serum-free DC medium for 45 min at 37°C as described previously (44). Then, DCs were plated in complete DC medium (10% FBS) at a concentration of 10^6^ cells/ml and incubated at 37°C for the desired time. Infections were stopped by centrifugation for 10 min at 400 × *g*, supernatants were collected and tested for cytokine production by a multiplex ELISA, and cell pellets were lysed for RNA isolation or protein analysis by Western blotting.

For NHBE cells, cells were washed 10 times with phosphate-buffered saline (PBS) prior to infection in order to remove mucins. An MOI of 0.2 was used, and the virus inoculum was prepared in PBS. After 45 min, the inoculum was removed, cells were washed one more time with PBS, and the PBS was removed so that the infection progressed in an air-liquid interphase. At the time points specified in Fig. 5, 200 μl of PBS was added over the infected cells and removed after a 15-min incubation at 37°C for subsequent replication assessment by plaque assay. Cells were lysed and kept at −80°C for RNA extraction and qRT-PCR analysis; also medium from the bottom chamber was collected and kept at −20°C to perform multiplex ELISAs. All of the NHBE cell infections were done in triplicates.

### Quantitative reverse transcription-PCR (qRT-PCR).

RNA from DCs and NHBE cells was extracted using an Agencourt RNAdvance Cell kit (version 2; Beckman Coulter) according to the manufacturer’s instructions. For infected DCs, expression of cellular and viral genes was quantified using iScript reverse transcription supermix (Bio-Rad) with oligo(dT) primers for the RT step and iQ SYBR green supermix (Bio-Rad) for the qPCR step, according to the manufacturer’s instructions. The PCR set up conditions were as follows: 95°C for 10 min and 40 cycles of 95°C for 10s and 60°C for 30s, followed by a melting curve. We used RPS11 as a housekeeping gene, and primers (forward and reverse) used for the quantification of the expression of each gene are summarized in Table 1. For the detection of viral segments of A/Puerto Rico/8/34 influenza virus, we used specific primers for M1, NP, HA, and NEP segments (Table 1). All the reactions were performed in duplicates. The efficiencies of the primers were approximately 100%. For vRNA detection, we used specific primers for the negative strand of NP according to a previously described method, with some modifications (77). Briefly, cDNA was obtained using a SuperScript IV first-strand synthesis system (Life Technologies) and a primer complementary to the negative strand of IAV with a non-IAV specific tail (5′-GGCCGTCATGGTGGCGAATATCGGAACTTCTGGAGGGGT-3′). PCR was carried out using iQ SYBR green supermix (Bio-Rad), and the following primers for detection: 5′-GGCCGTCATGGTGGCGAAT-3′ (forward; with the same sequence that the tail added during the RT reaction to improve specificity) and 5′-CTCAATATGAGTGCAGACCGTGCT-3′ (reverse) for vRNA. The relative mRNA expression level was calculated by the Δ*C_T_* method (*C_T_*_target_ − *C_T_*_housekeeping_), where *C_T_* is threshold cycle, as previously described (78), using RPS11 as the housekeeping gene to normalize the results (79).

**TABLE 1:** Sequences of specific primers used for detecting the expression of each gene

Gene	Forward primer (5′–3′)	Reverse primer (5′–3)
IFNA2	CTGAATGACTTGGAAGCCTG	ATTTCTGCTCTGACAACCTC
IFNB1	GTCAGAGTGGAAATCCTAAG	ACAGCATCTGCTGGTTGAAG
IFNL1	TGGGCTGAGGCTGGATACAG	TCTGGAGGCCACCGCTGACA
IFNL2	CGTGGGCTGAGGCTGGATAC	TGGCCCTGACGCTGAAGGTT
IFNL3	GGAGTAGGGCTCAGCGCATA	GCCTCCTCACGCGAGACCTC
TNFA	AGTGAAGTGCTGGCAACCAC	GAGGAAGGCCTAAGGTCCAC
IL-6	AATGCCAGCCTGCTGACGAA	CTGAGGTGCCCATGCTACAT
IP-10	TCCCATCACTTCCCTACATG	TGAAGCAGGGTCAGAACATC
IRF7	GGTGTGTCTTCCCTGGATAG	GCTCCAGCTCCATAAGGAAG
IFIT1	GACCTTGTCTCACAGAGTTC	TCGGAGAAAGGCATTAGATC
RANTES	TTGCCAGGGCTCTGTGACCA	AAGCTCCTGTGAGGGGTTGA
RPS11	GCCGAGACTATCTGCACTAC	ATGTCCAGCCTCAGAACTTC
ACTB	ACTGGAACGGTGAAGGTGAC	GTGGACTTGGGAGAGGACTG
TUBA1B	GCCTGGACCACAAGTTTGAC	TGAAATTCTGGGAGCATGAC
PR8 M1	TCAGGCCCCCTCAAAGCCGA	GGGCACGGTGAGCGTGAACA
PR8 NP	TACCTGCTTCTCAGTTCAAG	CAGCCTAATCAGACCAAATG
PR8 HA	TTGCTAAAACCCGGAGACAC	CCTGACGTATTTTGGGCACT
PR8 NEP	GGTTGATTGAAGAAGTGAGACACA	TCTCTTGCTCCACTTCAAGCA

To measure gene expression levels in NHBE cells, cDNA was synthesized from total RNA with AffinityScript Multi-Temp RT (Agilent Technologies). PCR Platinum Taq DNA polymerase and a buffer containing SYBR green (Life Technologies) were used for the PCR, which was performed in a thermocycler (ABI7900HT; Applied Biosystems). The housekeeping genes RPS11, TUBA1B, and ACTB were used as internal controls. All samples were run in triplicates and normalized to the median *C_T_* of the three corrected control genes in each sample, with the value converted to a nominal copy number per cell by assuming 2,500 copies of ACTB mRNA molecules per cell and an amplification efficiency of 93% for all reactions. Primer sequences can be found in Table 1.

### Western blot analysis.

At 6 and 12 hpi cells were lysed using 6 M urea buffer, and the total protein concentration was evaluated using a Bradford assay (Bio-Rad). The same amount of protein per sample was run using Laemmli loading buffer (Bio-Rad) in 4% to 20% SDS-polyacrylamide MiniPROTEAN TGX precast gels (Bio-Rad) under reducing conditions and then transferred to a polyvinylidene difluoride (PVDF) Immobilon transfer membrane (Millipore). Membranes were blocked in phosphate-buffered saline (PBS)–0.05% Tween 20 (Sigma-Aldrich) (PBS-T) containing 5% fat-free milk, washed 3 times with PBS-T, and incubated overnight (ON) at 4°C with the following antibodies diluted in PBS-T–5% fat-free milk: rabbit anti-PB2 (GTX125926; Genetex), rabbit anti-NP (45), rabbit anti-1-73NS1TX/98 (53), and mouse anti-β-actin (Sigma-Aldrich). The blots were washed and incubated with the corresponding secondary antibody, horseradish peroxidase (HRP)-linked sheep anti-mouse IgG, or donkey anti-rabbit (GE Healthcare). Antibody-protein complexes were detected using a Western Lighting chemiluminescent substrate (Thermo Scientific).

### Quantification of cytokine production by infected primary human DCs and NHBE cells.

Quantification of IFN-α, IFN-β, IFN-λ1, TNF-α, IL-6, IL-1β, and IP-10 secreted by human DCs after 12 h and by NHBE cells after 48 h of infection with different NS1-expressing recombinant viruses was performed with a multiplex ELISA kit (ProcartaPlex Assay; Invitrogen) according to the manufacturer’s recommendations. Data were analyzed by xPONENT software for the Luminex MAGPIx System.

### IFN-β reporter assay.

293T cells in 24-well plates were cotransfected using TransIT-Lt1 transfection reagent (Mirus), according to the manufacturer’s protocol, with 50 ng of IFN-β promoter expressing a firefly luciferase (FF-Luc) reporter plasmid (p125-FFLuc), 25 ng of *Renilla* luciferase (Ren-Luc) expression plasmid (pRL-TK; Promega), and 1, 5, or 25 ng of each pDZ-NS1 plasmid. Total plasmid DNA was kept constant with an empty expression vector. At 24 h posttransfection, cells were infected with a defective interfering (DI) particle-rich stock of Sendai virus (SeV) for 16 h to stimulate the IFN-β promoter activity.

### Imaging flow cytometry.

Human infected DCs were fixed with 1.6% paraformaldehyde (Electron Microscopy Science) for 10 min at 37°C, permeabilized with methanol (Sigma) for 10 min at 4°C, washed in PBS, and stained with mouse anti-M1/2 E10 PR8, rabbit anti-1-73NS1TX/98 (53), and DRAQ5 (Biostatus Limited) to label nuclei. Antibodies were conjugated with a Zenon antibody labeling kit to Alexa Fluor 405 (AF405) and AF488, (Z-25103 and Z-25302, respectively; Thermo Fisher Scientific) before staining. Following staining, single-cell images of each cell were acquired at a magnification of ×60 on an ImageStream Mark II flow cytometer (Amnis; EMD Millipore) and analyzed with IDEAS analysis software. Single-color controls were used for the creation of a compensation matrix that was applied to all files to correct for spectral cross talk. The “bright detail similarity” (BDS) feature of IDEAS was then used for measurement of the spatial correlation between the fluorescent signal emanating from Alexa Fluor 488-labeled NS1 and that of the nucleus and to obtain an NS1 nuclear localization score. The BDS score is the log-transformed Pearson’s correlation coefficient of the localized bright spots with a radius of three pixels or less within the masked area in the two input images (BDS score = ln[(1 + ρ)(1 − ρ)]). The higher the BDS score is, the higher is the level of colocalization. BDS scores of >0 were considered highly nuclear (Fig. 6B). This threshold for the BDS score was obtained by applying the similarity image analysis algorithm first to a negative set (bright-field image).

### Phylogenetic analysis.

NS1 protein sequences were aligned using the MUSCLE algorithm in MEGA7 (version 7.0.20). A phylogenetic tree was built with Geneious software (version 9.1.5) according to the neighbor joining clustering method and further visualized with FigTree.

### Statistical analysis.

Data sets were analyzed with GraphPad Prism software (GraphPad Software, Inc.). A two-way ANOVA followed by Tukey’s multiple-comparison test was used to determine significant differences in gene expression and cytokine production by DCs. Statistical significances between viral groups (human or avian NS1 viruses) in gene expression and cytokine production by NHBE cells were determined using a *t* test. Correlation analyses were performed using R, version 3.3.1, and Rstudio.
